# Atypical Tinea Corporis Revealing a Human Immunodeficiency Virus Infection

**DOI:** 10.7759/cureus.6551

**Published:** 2020-01-03

**Authors:** Joelle Brown, Matthew Carvey, Cristina Beiu, Robert Hage

**Affiliations:** 1 Medicine, St. George's University School of Medicine, St. George's, GRD; 2 Medicine, St. George’s University School of Medicine, St. George's, GRD; 3 Oncologic Dermatology, Elias Emergency University Hospital, Carol Davila University of Medicine and Pharmacy, Bucharest, ROU; 4 Otolaryngology, St. George's University School of Medicine, St. George's, GRD

**Keywords:** dermatophyte infections, hiv, immunodeficiency, tinea corporis

## Abstract

Dermatophytes are fungi that commonly cause superficial skin infections. While these rashes are typically benign and easily treated with topical antifungal medications, extensive presentations can indicate a more serious underlying immunodeficiency. We report on a teenage girl whose extensive rash led to a diagnosis of human immunodeficiency infection.

## Introduction

Dermatophytes, or tinea, are a type of fungi that infect keratinized tissues. These infections are categorized by their anatomical location and typically caused by one of three main species: Microsporum, Epidermophyton, or Trichophyton [1]. Tinea corporis, or “ringworm,” refers to an infection usually on the torso and the limbs, typically caused by the Trichophyton species and acquired through one or more of the following routes: contact with infected humans or animals, exposure to contaminated soil, or exposure to fomites. It is characterized by an annular plaque with advancing, raised erythematous, scaling borders surrounding a clear center [2]. Dermatophyte infections are common worldwide; however, unusually severe cases have a higher prevalence among immunocompromised patients and warrant further investigation [3].

## Case presentation

**Figure 1:**
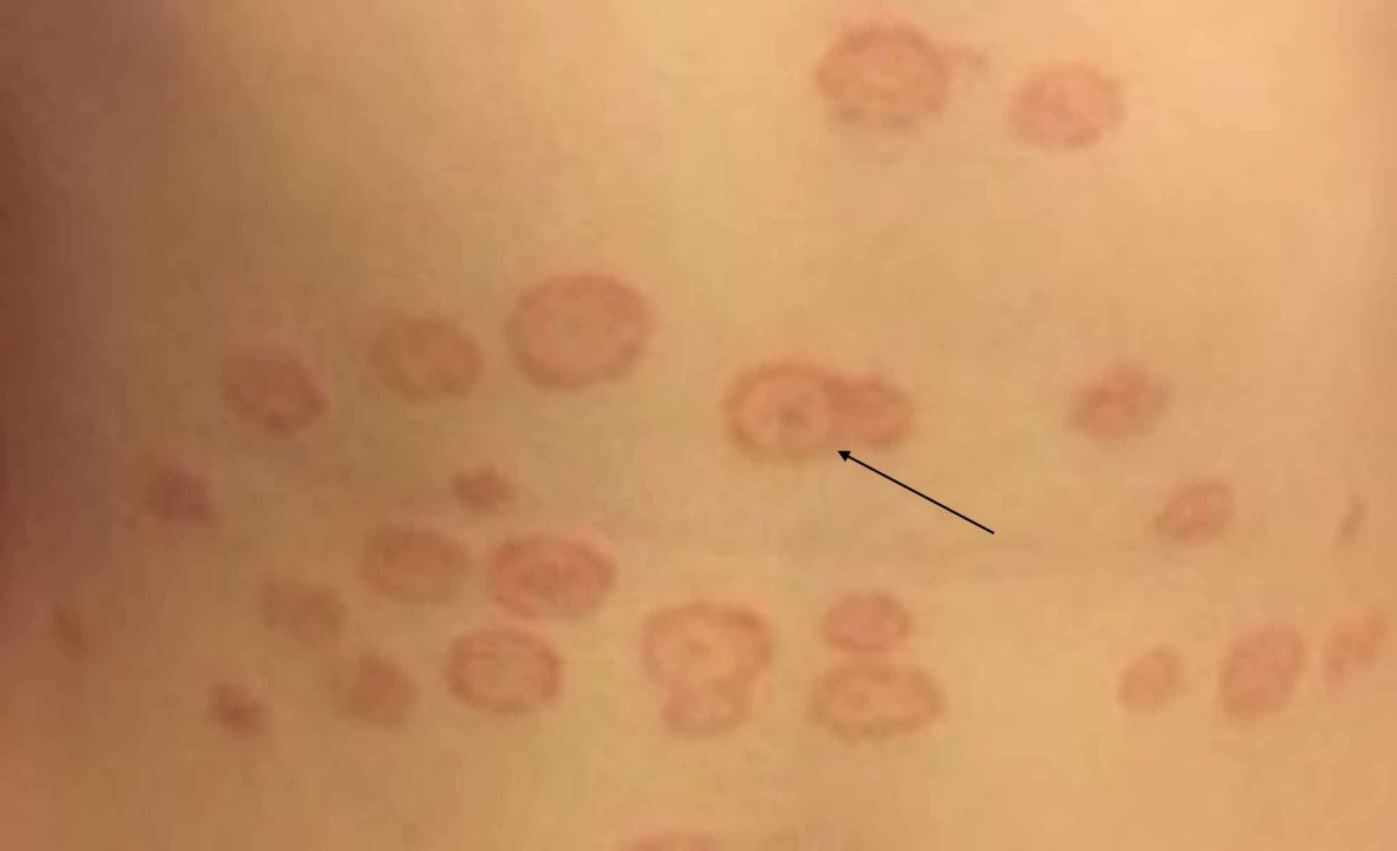
Annular, erythematous lesions on the torso with central clearing and raised scaly borders; some lesions have concentric, “targetoid” appearance (black arrow).

**Figure 2:**
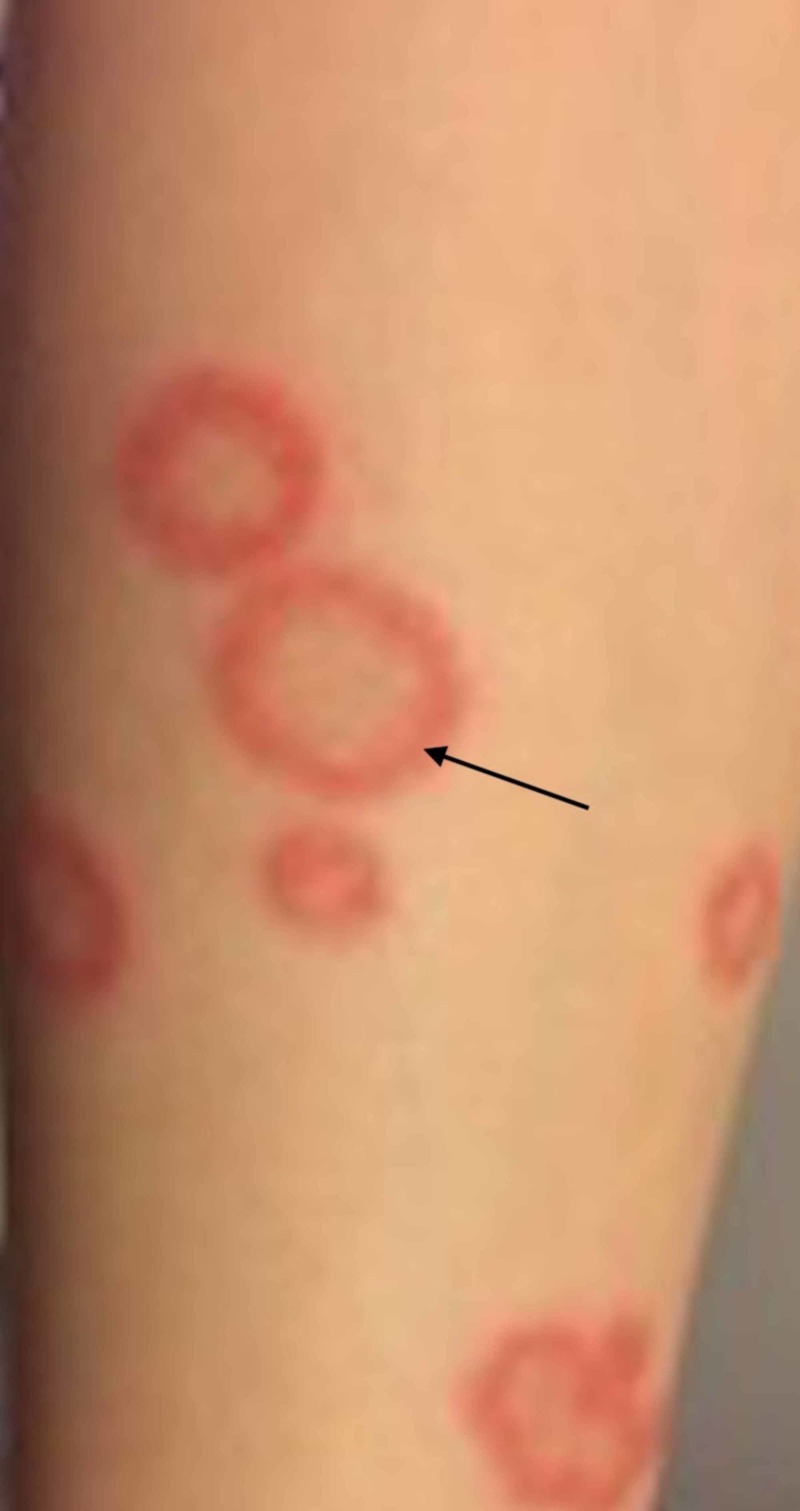
Ring-shaped and polycyclic plaques on the legs. The active borders (black arrow) indicate the centrifugal spread of the rash.

A teenage girl presented to the Elias Emergency University Hospital dermatology department for the assessment of a skin rash consisting of multiple annular, erythematous, slightly scaly plaques, including a few targetoid appearing lesions on her torso (Figure 1) and legs (Figure 2). Medical history was significant for recurrent infections with herpes simplex virus type 1 (HSV-1).

The patient history revealed that she had presumptively been diagnosed with erythema multiforme in another hospital. She was prescribed topical therapy with a medium potency corticosteroid cream. Despite compliance with treatment, the rash persisted, which prompted the patient to present to the Elias Emergency University Hospital for a second opinion. Physicians there performed a potassium hydroxide (KOH) preparation that revealed segmented hyphae. This led to a diagnosis of tinea corporis. The patient was prescribed terbinafine 250 mg/day for one week, which successfully cleared her rash; however, the extensive presentation of the infection on her trunk and legs was unusual and prompted further examination. Subsequent testing revealed a positive diagnosis for human immunodeficiency (HIV) infection.

## Discussion

Dermatophytes commonly cause superficial skin infections in both immunocompetent and immunocompromised patients. In the United States, tinea is the second most commonly reported skin infection overall after acne [4]. Tinea corporis is particularly prevalent among immunocompromised patients. In a study by Kaviarasan et al., a diagnosis of dematophytosis was made in 41 out of 185 HIV-positive patients (22.2%), with tinea corporis being the most common infection seen in 22 (53.7%) cases [3]. Tinea corporis typically presents as distinct red, outward-spreading papules that eventually coalesce into scaly papules and plaques on smooth, bare skin. As the lesions advance, they developed raised borders and their centers may clear, leaving a hypopigmented central area that has a targetoid appearance [4]. In immunocompromised patients, however, the clinical presentation can be more extensive, involving a greater body surface area as was seen in our patient. Immunocompromised patients are also more prone to develop deep dermatophytosis, a rare condition characterized by dermatophyte invasion of the dermis and hypodermis instead of just the keratinized skin [5].

The pathogenesis for dermatophyte infection is shared among tinea species. Infection starts with the deposition and adherence of fungal spores to the outer surface of the skin. It is hypothesized that dermatophyte-specific proteases mediate adherence to keratinocytes. Dermatophytes then secrete keratinases to penetrate the stratum corneum at a faster rate than desquamation occurs. The diffusion of fungal metabolic products through the stratum basale results in the classic appearance of scaling, annular, itchy patches that spreads centrifugally [6]. Clearance of a dermatophyte infection is typically via a cell-mediated immune response through the release of inflammatory cytokines such as interferon gamma (IFN-ɣ) from Th1/Th17 cells [7]. Dysregulation of the immune system that results in a stronger Th2, or humoral response, and produces elevated IgE and IgG4 antibodies could be responsible for atypical presentations or more chronic infections [7]. Deficiency in caspase recruitment domain containing protein 9 (CARD9) has also been shown to be associated with more severe presentations of tinea infections, even in non-HIV individuals. CARD9 is a member of the caspase protein family integral to the process of cell apoptosis. Its expression on myeloid cells helps with the recognition of fungal pathogen-associated molecular patterns, and production of IL-17 and Th17 cells [5]. The importance of the cell-mediated immune response in eliminating dermatophyte infections could explain why those with dysfunctional cell-mediated immunity, such as patients with HIV and AIDS, have more severe presentations of these infections.

Important differential diagnoses for this case include erythema multiforme, Candida albicans infection, and Pityrosporum versicolor infection. Erythema multiforme is a rash characterized by polymorphous eruption of macules, papules, and “target” lesions. Unlike tinea corporis, these target lesions have a “dusky” central zone, a pale pink edematous zone, and a peripheral red ring. They are symmetrically distributed with a propensity for the distal extremities. It is commonly associated with recurrent HSV infections. Like tinea corporis, its pathogenesis is driven by cell-mediated immune response in which CD4+ cells respond to viral antigens by producing IFN-ɣ leading to an inflammatory cascade. Erythema multiforme is usually asymptomatic, and typically resolves spontaneously. Corticosteroid therapy can provide symptomatic relief, and antiviral treatment with acyclovir or valcyclovir can prevent recurrence. Skin biopsy shows mononuclear cell infiltrates, dermal edema, or necrotic keratinocytes, depending on the stage and zone of the lesion from which the biopsy was taken; segmented hyphae, as was seen in our patient, would not be present [8]. Thus, erythema multiforme is an unlikely diagnosis.

C. albicans is a yeast commonly found on skin and responsible for superficial infections, particularly in those with underlying immunodeficiencies. Candida skin infections, however, present with a thickening of skin, hyperkeratosis, and erythema [9]. Lesions tend to have an irregular shape with satellite lesions at the edge of inflamed areas [4]. Under microscopy, C. albicans shows ovoid-shaped budding yeasts, elongated ellipsoid cells with constrictions at the septa, or pseudohyphae, or as parallel-walled true hyphae [10]. The lesions on our patient were consistently a targetoid shape with no hyperkeratosis, and microscopy showed segmented hyphae, ruling out a C. albicans infection.

Pityrosporum versicolor, or Tinea versicolor, is another common superficial fungal skin infection. Despite its name, it is caused by the fungus Malassezia furfur and is not a dermatophyte infection [4]. It presents as a rash of round or oval macules that can be pruritic and hypopigmented, hyperpigmented, or erythematous. It is typically a chronic condition that is more prevalent in warm weather and affects the upper back/chest, arms, or face, though case reports have described it appearing on the legs and in the groin [4,11]. Under KOH preparation, it shows spores and short hyphae, but not the segmented hyphae seen in our patient [11]. Thus, the clinical and microscopic characteristics of our patient’s infection make tinea corporis the most likely pathogen.

## Conclusions

In this young patient, extensive presentation of a cutaneous infection proved to be an early sign of HIV infection. This illustrates how important it is for clinicians to maintain a high index of suspicion when evaluating atypical presentations of common fungal infections, as any skin changes always constitute potential markers of internal disease.
